# Factors Associated with Post-Acute Sequelae of SARS-CoV-2 (PASC) After Diagnosis of Symptomatic COVID-19 in the Inpatient and Outpatient Setting in a Diverse Cohort

**DOI:** 10.1007/s11606-022-07523-3

**Published:** 2022-04-07

**Authors:** Sun M. Yoo, Teresa C. Liu, Yash Motwani, Myung S. Sim, Nisha Viswanathan, Nathan Samras, Felicia Hsu, Neil S. Wenger

**Affiliations:** Department of Medicine, David Geffen School of Medicine, University of California, Los Angeles, 757 Westwood Plaza, Los Angeles, CA 90095 USA

## Abstract

**Background:**

The incidence of persistent clinical symptoms and risk factors in Post-Acute Sequelae of SARS-CoV-2 (PASC) in diverse US cohorts is unclear. While there are a disproportionate share of COVID-19 deaths in older patients, ethnic minorities, and socially disadvantaged populations in the USA, little information is available on the association of these factors and PASC.

**Objective:**

To evaluate the association of demographic and clinical characteristics with development of PASC.

**Design:**

Prospective observational cohort of hospitalized and high-risk outpatients, April 2020 to February 2021.

**Participants:**

One thousand thirty-eight adults with laboratory-confirmed symptomatic COVID-19 infection.

**Main Measures:**

Development of PASC determined by patient report of persistent symptoms on questionnaires conducted 60 or 90 days after COVID-19 infection or hospital discharge. Demographic and clinical factors associated with PASC.

**Key Results:**

Of 1,038 patients with longitudinal follow-up, 309 patients (29.8%) developed PASC. The most common persistent symptom was fatigue (31.4%) followed by shortness of breath (15.4%) in hospitalized patients and anosmia (15.9%) in outpatients. Hospitalization for COVID-19 (odds ratio [OR] 1.49, 95% [CI] 1.04–2.14), having diabetes (OR, 1.39; 95% CI 1.02–1.88), and higher BMI (OR, 1.02; 95% CI 1–1.04) were independently associated with PASC. Medicaid compared to commercial insurance (OR, 0.49; 95% CI 0.31–0.77) and having had an organ transplant (OR 0.44, 95% CI, 0.26–0.76) were inversely associated with PASC. Age, race/ethnicity, Social Vulnerability Index, and baseline functional status were not associated with developing PASC.

**Conclusions:**

Three in ten survivors with COVID-19 developed a subset of symptoms associated with PASC in our cohort. While ethnic minorities, older age, and social disadvantage are associated with worse acute COVID-19 infection and greater risk of death, our study found no association between these factors and PASC.

**Supplementary Information:**

The online version contains supplementary material available at 10.1007/s11606-022-07523-3.

## INTRODUCTION

As millions of people recover from COVID-19 amidst the global pandemic, many continue to report an array of persistent symptoms after infection, termed post-acute sequelae of SARS-CoV-2 (PASC). Commonly reported PASC symptoms range from fatigue and dyspnea to “brain fog” and anosmia, with ongoing disability and disruption of work, social, and home lives.^1–4^ Centers for Disease Control and Prevention classify the array of symptoms lasting 4 or more weeks after COVID-19 infection as a Post-COVID Condition, which may include long-haul COVID, long COVID, or PASC. Most efforts to describe PASC and factors associated with PASC have focused on hospitalized adult patients^1,2,4^ and more recently on patient with mild COVID-19 infection treated in the outpatient setting up to 9 months after infection.^5,6^ Although there are studies that have looked at factors associated with PASC,^7^ there are no prospective cohort studies to our knowledge that have evaluated the association of ethnicity, social vulnerability, and insurance status with developing PASC. Although COVID-19 has disproportionately impacted racial and ethnic minority groups, previously studied PASC cohorts in the USA have small proportions of Latinx, Black or African American, and Asian American and Pacific Islander (AAPI) patients.^5^

In April 2020, University of California Los Angeles (UCLA) Health created a COVID Ambulatory Monitoring Program to clinically care for the diverse group of COVID-19 patients discharged from the hospital as well as high-risk COVID-19 patients cared for in the outpatient setting. Patients were eligible from all parts of the UCLA Health system, which includes more than 40 primary care clinics and 20 UCLA Hospitalist services in hospitals across southern California. This COVID-19 program collected standardized clinical data to guide the approach to monitoring and care. Using these patient-reported data combined with clinical information from the electronic health record (EHR), we describe a population-based cohort of patients with symptomatic COVID-19, characterize the timeline of persistent clinical symptoms, and identify factors associated with PASC.

PASC terminology is in evolution and can include a wide range of clinical manifestations including psychiatric manifestations and evidence of organ dysfunction as a result of SARS-CoV-2, including new symptoms or clinical findings that were not evident at the time of acute COVID-19 infection. For our evaluation, we focused on a subset of symptoms associated with PASC as described in the Clinical and Functional Survey (available in Appendix), which we will reference as PASC in our paper.

## METHODS

A longitudinal, prospective cohort of adults with laboratory confirmed SARS-CoV-2 infection was enrolled in the UCLA COVID Ambulatory Program starting April 2020. Standardized follow-up was performed to monitor patients with COVID-19 discharged from the two UCLA hospitals, UCLA patients discharged from 20 other local hospitals, and UCLA outpatients who were referred by their primary care providers. COVID-19 patients discharged from the general ward services were approached post-discharge to enroll them in the program. Outpatients with new infections deemed clinically high risk by their primary care providers were referred for enrollment. A questionnaire administered by nurses over the telephone collected information on functional status prior to COVID-19 infection and post-COVID clinical symptoms. Follow-up monitoring continued at 30, 60, and 90 days after hospital discharge for post-discharge patients or date of positive COVID-19 test for outpatients to evaluate for persistent symptoms. A multidisciplinary team of primary care physicians and specialists followed patients to address persistent symptoms or clinical deterioration. The study was approved by the UCLA institutional review board (IRB#20-001358).

A monitoring questionnaire (available in the Appendix) assessed whether the patient felt that his or her health was back to normal. The survey queried baseline function by asking about maximal exertion level prior to COVID-19 infection: vigorous activities such as running, lifting heavy objects, and participating in strenuous sports; moderate activities, such as moving a table, pushing a vacuum cleaner, bowling, or playing golf; climbing one flight of stairs; walking one block; lifting or carrying groceries; bathing or dressing yourself.^8^ Functional limitation over the past 4 weeks was assessed using this item during each survey. Perceived cognitive deficits were evaluated with three questions modified from the Perceived Deficits Questionnaire-5^9^ that ask whether patients in the last 4 weeks had trouble getting things organized, had trouble concentrating on things, or forgetting what the patient talked about after a telephone conversation. Lastly, patients were asked about the following symptoms over the past 4 weeks: fever, chills or night sweats; loss of smell or taste; fatigue; shortness of breath; chest pain; numbness or tingling; nausea, vomiting, or diarrhea; muscle aches; and rash.

Demographic characteristics (age, gender, race, ethnicity) were obtained from the EHR, as were a history of diabetes mellitus or organ transplant, body mass index (BMI), Elixhauser comorbidity index,^10^ and level of medical care required for the initial COVID-19 illness (ambulatory care, emergency room, hospital, and intensive care unit [ICU]). Insurance was collapsed into commercial, Medicare, Medicaid, and none/other. Social Vulnerability Index (SVI) was calculated and split into quartiles.^11^

Baseline demographic characteristics, functional status, and clinical characteristics were evaluated in the full cohort and compared among patients treated initially in the outpatient setting, in the inpatient setting, and in the ICU. Patients were characterized as having PASC if they noted persistent COVID-19 symptoms on the 90-day post-discharge survey (or the 60-day survey if the 90-day survey was incomplete). Survey attrition flow diagram is included in the Appendix (Supplemental Figure 1). Baseline characteristics of PASC patients in the inpatient and outpatient settings were compared to patients without persisting post-acute symptoms. Among patients with PASC, symptoms were compared at the time of acute infection, 30 days, 60 days, and 90 days, presented separately for patients with PASC who received their initial COVID-19 treatment in the inpatient versus outpatient setting.

### Statistical Analysis

Summary statistics (i.e., mean, standard deviation SD, and percentage) for demographics and clinical characteristics are reported for the full cohort. We performed *t*-tests for a difference in means between groups for age, BMI, and Elixhauser Comorbidity Index and used chi-square tests to test differences in proportions between groups for sex, race/ethnicity, diabetes, transplant, payor status, and baseline functional status. We used a multivariable logistic regression model to evaluate the factors associated with the development of PASC. The pre-specified factors included in the model were demographics (age, sex, race), clinical characteristics (diabetes, BMI, transplant status), insurance type, SVI, COVID-19 care venue, and baseline function. We performed 5 multiple imputations (MI) for Elixhauser (6.0% missingness, and 10.3% unknown) and performed logistic regression analysis for each imputed set. We did not include Elixhauser in the final logistic regression model after finding that it was not statistically significant in those MI sets and the inclusion of imputed Elixhauser in the model did not affect the estimates of odds ratios (ORs) of other factors. In order to further characterize clinical factors associated with PASC, we disaggregated the components of the Elixhauser index and evaluated their individual relationships with PASC using chi-square and Fisher’s exact tests. Factors included in the final model were selected regardless of their statistical significance considering the clinical importance and to examine the effect of socioeconomic status on the PASC outcome. Two-sided *P* value <0.05 was considered statistically significant and analyses were done using SAS 9.4 (Cary, NC).

## RESULTS

### Participant Characteristics

Of the 1,296 enrolled in the program between April 2020 to February 2021, 1,038 patients (80.1%) completed follow-up surveys at 30, 60, or 90 days after hospital discharge or outpatient diagnosis and were included in the study. Of the 800 patients treated for COVID-19 in the hospital, 152 (19%) received treatment in the ICU. Of the 238 patients treated as outpatients, 36 (15.1%) received care in the emergency department. The mean age of the cohort was 60 years (interquartile range, 37 to 83) with an even split by gender overall, but more women receiving outpatient care. Thirty percent were White and 42% Latinx. More than one-third of the cohort had diabetes, over 10% had received a solid organ transplant, and mean BMI was nearly 30. Forty-two percent of patients had commercial insurance and mean SVI was 0.46 (interquartile range 0.16 to 0.76). At baseline, most patients could complete vigorous or moderate activities. Men, patients with diabetes, and Latinx patients were more likely to have been admitted to the hospital or the ICU (Table 1).

**Table 1:** Demographics and Clinical Characteristics of COVID-19 Patients

	Full cohort (*n*=1,038)	Outpatient (*n*=238)	Inpatient (non-ICU) (*n*=648)	Inpatient ICU (*n*=152)
Age, mean (IQR)	60 (37–83)	60 (36–84)	60 (38–83)	60 (41–79)
Sex, *N* (%)				
Women	515 (49.6)	147 (61.8)^a,b^	309 (47.7)^a^	59 (38.8)^b^
Men	523 (50.4)	91 (38.2)^a,b^	339 (52.3)^a^	93 (61.2)^b^
Race/ethnicity, *N* (%)				
White	317 (30.5)	94 (39.5)^a,b^	194 (29.9)^a^	29 (19.1)^b^
Hispanic or Latino	432 (41.6)	74 (31.1)^a,b^	266 (41.0)^a^	92 (60.5)^b^
Black	74 (7.1)	15 (6.3)	50 (7.7)	9 (5.9)
Asian	82 (7.9)	14 (5.9)	58 (9.0)	10 (6.6)
Other race	85 (8.2)	15 (6.3)	60 (9.3)	10 (6.6)
Unknown	48 (4.6)	26 (10.9)^a,b^	20 (3.1)^a^	2 (1.3)^b^
Diabetes, *N* (%)	393 (37.9)	61 (25.6)^a,b^	252 (38.9)^a^	80 (52.6)^b^
Organ transplant recipient, *N* (%)	111 (10.7)	16 (6.7)	77 (11.9)	18 (11.8)
Body Mass Index, mean (SD)	29.9 (7.1)	29.4 (7.1)	30.0 (7.2)	30.3 (6.8)
Elixhauser Comorbidity Index, mean (SD)	10.6 (12.3)	8.7 (11.9)^b^	10.7 (12.2)	13.0 (13.0)^b^
SVI, mean (SD)	0.46 (0.3)	0.39 (0.29)^a,b^	0.48 (0.3)^a^	0.56 (0.3)^b^
Payor, *N* (%)				
Commercial	438 (42.2)	141 (59.2)^b^	244 (37.7)	53 (34.9)^b^
Medicare	382 (36.8)	85 (35.7)	243 (37.5)	54 (35.5)
Medicaid	175 (16.9)	7 (2.9)^a,b^	128 (19.8)^a^	40 (26.3)^b^
Other or none	43 (4.1)	5 (2.1)^a^	33 (5.2)^a^	5 (3.3)
Baseline functional status, *N* (%)				
Vigorous activities	236 (22.7)	61 (25.6)	140 (21.6)	35 (23.0)
Moderate activities	506 (48.7)	137 (57.6)^a,b^	303 (46.8)^a^	66 (43.4)^b^
Climb 1 flight of stairs	68 (6.6)	8 (3.4)	48 (7.4)	12 (7.9)
Walk 1 block	149 (14.4)	20 (8.4)^a,b^	102 (15.7)^a^	27 (17.8)^b^
Carry groceries	6 (0.6)	0	6 (0.9)	0
Bathe or dress	44 (4.2)	8 (3.4)	29 (4.5)	7 (4.6)
Missing	29 (2.8)	4 (1.7)	20 (3.1)	5 (3.3)

^a^*p* < .01 for comparison of patients with SARS-CoV-2-positive tests treated in the outpatient setting compared to those treated inpatient (non-ICU)

^b^*p* < .01 for comparison of patients with SARS-CoV-2-positive tests treated in the outpatient setting compared to those treated in the ICU

### Persistent Symptoms

Of the 1038 patients, 309 (29.8%) reported persistent symptoms on the follow-up survey at least 60 days after the acute illness, defined as PASC in this cohort. Of the 800 patients who received treatment for COVID-19 in the hospital, 246 (30.8%) developed PASC whereas 63 (26.5%) of the 238 treated as high-risk outpatients developed PASC.

**Figure 1:**
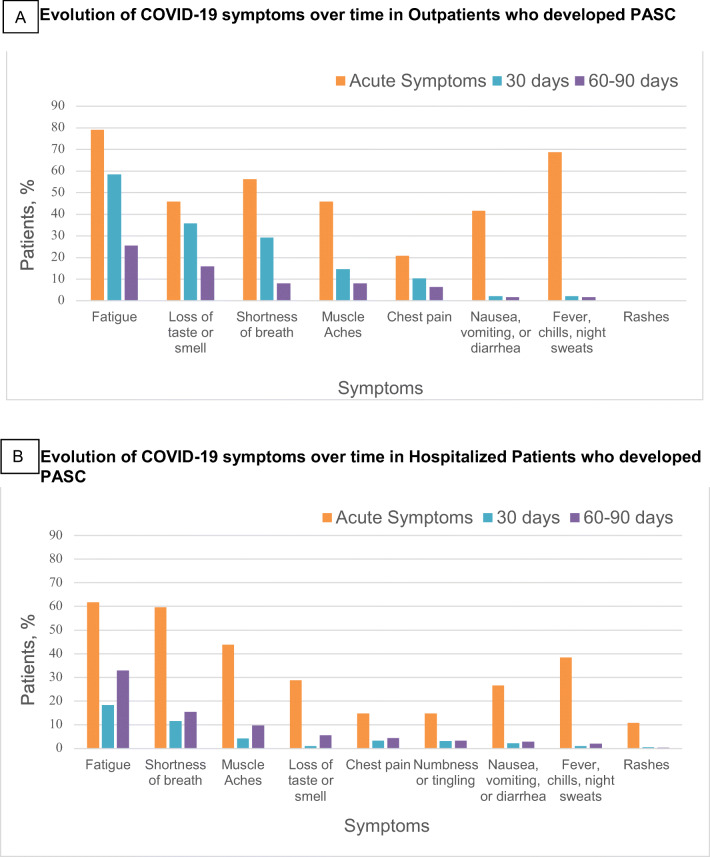
Symptoms described by patients with PASC who received COVID-19 care in the outpatient (**a**) or inpatient (**b**) settings. Symptoms reported refer to the acute phase of illness, 30 days after discharge or diagnosis, and 60–90 days after discharge or diagnosis. Acute symptoms and symptoms persistent at 30 days were reported in the 30-day survey (*N*=849), and symptoms persistent 60+ days were reported in the 60-day or 90-day survey (*N*=879).

Symptoms during the acute period of COVID-19 illness were reported on the 30-day survey. Of the PASC patients who completed the 30-day survey (*n*=231), the most commonly reported symptoms were fatigue in 169 patients (73.2%) followed by shortness of breath in 147 patients (63.6%), fevers and chills in 119 (51.5%), and muscle aches in 117 patients (50.6%). In terms of persistent symptoms at least 60 days after infection, fatigue was the most commonly reported symptom (31.4%), followed by shortness of breath (13.9%), and loss of taste or smell (9.8%). Persistent fever (1.9%) and rash (< 1%) were rare. When comparing hospitalized to outpatient PASC patients, fatigue was the most common persistent symptom in both groups. The next most common symptom in hospitalized patients was shortness of breath (15.4%), whereas it was loss of taste or smell (15.9%) in outpatients (Figure 1A, B).

### Factors Associated with PASC

In bivariate analyses, factors associated with PASC were different among patients treated for COVID-19 in the inpatient versus outpatient setting. In the outpatient setting, PASC patients were younger, more likely to be White, women, and commercially insured. In the inpatient setting, age and race were unrelated to PASC, and women were more likely to report symptoms consistent with PASC. In both settings, transplant patients were less likely to develop PASC and baseline functional status prior to COVID-19 infection was not related (Table 2).

**Table 2:** Demographics and Characteristics of COVID-19 PASC Cohort and Non-PASC Cohort by Highest Level of Care

	PASC outpatient	Non-PASC outpatient	PASC inpatient	Non-PASC inpatient
*N*	63	175	246	554
Age, mean (IQR)	56.6 (43–70) ^a^	61.4 (50–72) ^a^	60.0 (50–71)	59.9 (49–71)
Sex, *N* (%)				
Women	42 (66.7)	105 (60.0)	123 (50.0)	245 (44.2)
Men	21 (33.3)	70 (40.0)	123 (50.0)	309 (55.8)
Race/ethnicity, %				
White	28 (44.4)	66 (37.7)	71 (28.9)	152 (27.4)
Hispanic or Latino	17 (27.0)	57 (32.6)	112 (45.5)	246 (44.4)
Black	4 (6.3)	11 (6.3)	18 (7.3)	41 (7.4)
Asian	4 (6.3)	10 (5.7)	19 (7.7)	49 (8.8)
Other race	4 (6.3)	11 (6.3)	20 (8.1)	50 (9.0)
Unknown	6 (9.5)	20 (11.4)	6 (2.4)	16 (2.9)
Comorbidities, %				
Diabetes	15 (23.8)	46 (26.3)	113 (45.9)^b^	219 (39.5)^b^
Transplant	1 (1.6)^a^	15 (8.6)^a^	21 (8.5)^b^	74 (13.4)^b^
BMI, mean (SD)	30.4 (7.7)	29.1 (6.8)	31.0 (7.6)^b^	29.7 (6.9)^b^
Elixhauser Comorbidity Index, mean (SD)	7.6 (10.6)	9.1 (12.3)	10.0 (12.6)^b^	11.7 (12.3)^b^
SVI, mean (SD)	0.35 (0.28)	0.4 (0.29)	0.49 (0.31)	0.49 (0.3)
Payor, %				
Commercial	42 (66.7)	99 (56.6)	100 (40.7)	197 (35.6)
Medicare	19 (30.2)	66 (37.7)	95 (38.6)	202 (36.5)
Medical	0	7 (4.0)	40 (16.3)^b^	128 (23.1)^b^
Other or none	2 (3.2)	3 (1.7)	11 (4.5)	27 (4.9)
Baseline functional status, *N* (%)				
Vigorous activities	22 (34.9) ^a^	39 (22.3) ^a^	53 (21.5)	122 (22.0)
Moderate activities	31 (49.2)	106 (60.6)	120 (48.8)	249 (44.9)
Climb 1 flight of stairs	2 (3.2)	6 (3.4)	18 (7.3)	42 (7.6)
Walk 1 block	5 (7.9)	15 (8.6)	34 (13.8)	95 (17.1)
Carry groceries	0	0	1 (0.4)	5 (0.9)
Bathe or dress	3 (4.8)	5 (2.9)	12 (4.9)	24 (4.3)
Missing	0	4 (2.3)	8 (3.3)	17 (3.1)

^a^*p* < .01 for comparison of PASC patients compared to non-PASC patients treated in the outpatient setting

^b^*p* < .01 for comparison of PASC patients compared to non-PASC patients treated in the inpatient setting

**Figure 2:**
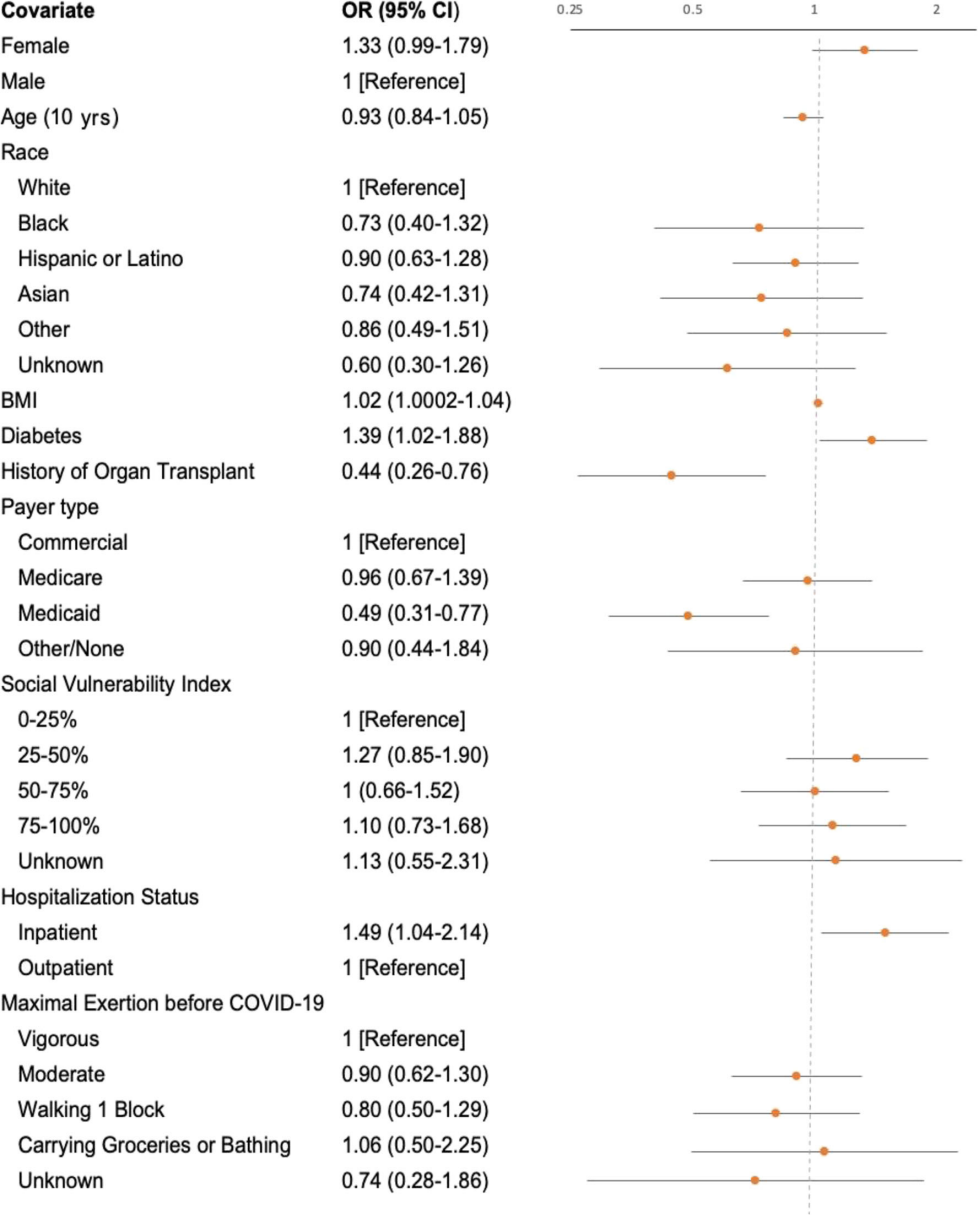
Multivariate logistic regression for assessing factors associated with PASC. Scale for the OR in Figure 2 is represented on a log scale.

In adjusted analyses, hospitalization for COVID-19 (OR 1.49, 95% confidence interval [CI] 1.04–2.14), having diabetes (OR, 1.39; 95% CI 1.02–1.88), and higher BMI (OR, 1.02; 95% CI 1.0002–1.04) were independently associated with developing PASC. Having Medicaid compared to commercial insurance (OR, 0.49; 95% CI 0.31–0.77) or a history of organ transplant (OR 0.44, 95% CI, 0.26–0.76) was inversely associated with developing PASC. Age, race/ethnicity, SVI, and baseline functional status were not associated with developing PASC (Fig. 2).

## DISCUSSION

In this prospective cohort of individuals with COVID-19 infection, 30% of patients developed a subset of symptoms associated with PASC. This large, diverse cohort achieved a longitudinal follow-up of 80% of patients after the acute COVID-19 illness. Studying a diverse population treated in a single health system, we are able to control for factors such as access and quality of care often lacking in existing studies.^5,12^ This health system-based COVID-19 population reveals the startling findings that age, race, and economic disadvantage appear unassociated with development of PASC. This contrasts with COVID-19 infection rates, hospitalizations, and deaths that are disproportionately higher in racial and ethnic minority communities and older people.^13,14^

Existing prospective studies have not evaluated race and ethnicity and its association with PASC. Sudre et al. found increasing age, female gender, hospitalization, and more than five symptoms in the first week of illness to be associated with PASC, but were unable to analyze the impact of ethnicity due to incomplete data.^7^ Other studies have described hospitalized patients as more likely to be older, not White, and with lower household incomes compared to outpatients,^12^ but evaluation of the association between race/ethnicity, SVI, insurance, and PASC were not done.

Current data indicate that ethnic minorities represent a disproportionate number of COVID-19 cases, hospitalizations, and deaths in the USA.^13–15^ Ethnic minorities in the USA diagnosed with COVID-19 were likely to have a greater number of underlying clinical comorbidities,^16^ lower socioeconomic status,^17^ loss of health insurance during the pandemic,^18,19^ and poor access to healthcare. In cohort studies of patients hospitalized for COVID-19, race and ethnicity were not independently associated with in-hospital mortality after adjusting for factors such as age, sex, comorbidities, insurance, and neighborhood deprivation.^16,20^ This suggests that access to care is an important factor in COVID-19 outcomes. In our study, race and ethnicity was not associated with developing PASC. One possible explanation is that patients had access to the same health system with standardized follow-up. Another possible explanation is that factors that contribute to risks of contracting COVID-19 are not as important in the COVID-19 recovery process and development of PASC. Another factor to consider is whether report of symptoms and expectations for recovery differ across socioeconomic, ethnic, and racial groups and whether the tools used to detect PASC equitably capture these reports.

Existing literature suggests that women were more likely than men to develop PASC,^7^ whereas our data found a non-statistically significant trend in this direction. It has been suggested this may be due to differences in immunity with a higher prevalence of autoimmune disease in women.^21^ Diabetes and elevated BMI were associated with developing PASC, which may be due to increased inflammation seen in SARS-CoV-2^22^ potentially leading to microvascular and macrovascular complications.^23,24^ Interestingly, surviving transplant patients were less likely to develop PASC. The dampened host inflammatory response to COVID-19 for those on immunosuppression agents may play a role, as suggested by a prior study showing solid organ transplant recipients had a faster decline in disease severity over time.^25^

Surprisingly, patients with commercial insurance had double the likelihood of developing PASC compared to patients with Medicaid. This association will be important to explore further to understand if insurance status in this group is representing unmeasured demographic factors or exposures.

In the acute period of illness, the most commonly reported clinical symptoms among patients who fit our PASC definition were fatigue then shortness of breath, in line with existing studies.^1,2^ Although fatigue was the most common persistent symptom in hospitalized and non-hospitalized patients, this was followed by loss of sense of smell in outpatients and shortness of breath in inpatients, which is in line with previous studies.^3,5^ This variation in symptoms at presentation and over time suggests differences in the clinical phenotypes of those with mild to moderate COVID-19 treated in the outpatient setting compared to those with severe COVID-19 requiring hospitalization.

Strengths of our study include a large diverse cohort of COVID-19 patients prospectively followed in a single health system with a large number of organ transplant patients, and standardized longitudinal data to assess symptom evolution over time. Study limitations include potential bias from subjective rating of symptoms and functional status, evaluation of a limited subset of symptoms encapsulated by PASC, not having a comparator group of patients with persistent symptoms after non-COVID hospital admissions, and limited information about pre-existing conditions in our patient population. In addition, survivorship bias may exist where the analysis was limited to individuals that survived to at least 30 days after COVID-19 diagnosis, and referral bias in the outpatient cohort, as only patients deemed clinically high risk were referred to the program, which may affect the generalizability in the outpatient cohort.

## CONCLUSION

In this diverse prospective cohort of symptomatic COVID-19 patients treated in the inpatient and outpatient setting, nearly one-third developed a subset of symptoms associated with PASC. The most common symptoms that persisted were different in outpatients compared to inpatients, with fatigue and anosmia in outpatients and fatigue and dyspnea in inpatients. Finally, the lack of relationship between factors related to more serious COVID-19 illness (age, ethnicity, baseline function, and socioeconomic vulnerability) suggests that the long-term effects of COVID-19 may vary from those producing acute illness. These findings, along with variation by insurance status and the protective nature of transplantation, should stimulate additional study to understand the pathophysiologic factors underlying PASC, as well as the tools and methods used to detect PASC. Understanding the effects of long COVID will allow for more effective education among patients and providers, and allow for appropriate healthcare resource utilization in the evaluation and treatment of PASC.

## Supplementary Information

ESM 1 (DOCX 53 kb)
