# Supplementary material for: Factors Associated with Post-Acute Sequelae of SARS-CoV-2 (PASC) After Diagnosis of Symptomatic COVID-19 in the Inpatient and Outpatient Setting in a Diverse Cohort
Source: J Gen Intern Med. 2022 Apr 7;37(8):1988–95. doi: 10.1007/s11606-022-07523-3 (PMC8989256; doi:10.1007/s11606-022-07523-3)
Supplement: Supplementary file 1 — (DOCX 53 kb) [file 11606_2022_7523_MOESM1_ESM.docx]

**APPENDIX**

# CLINICAL AND FUNCTIONAL SURVEY

Information obtained by trained clinical staff (RNs) at 30 days, 60 days, and 90 days after acute illness or hospitalization.

| **1** | Do you feel your health is back to normal?  (If YES, skip next question 3) | ‌ Yes  ‌ No |
| --- | --- | --- |
| **2** | Compare to 4 weeks ago, how would you rate your health now? (1=way worse than prior baseline; 10=back to normal) |  |
| **3** | During the past 4 weeks, has your physical health interfered with your normal social activities with family, friends, neighbors, or groups? | ‌ Yes  ‌ No   Explain: |
| **4** | During the past 4 weeks, have you had any problems with your work or other regular daily activities as a result of your physical health? | ‌ Yes  ‌ No   Explain: |
| **5** | What is the maximal exertion you could do BEFORE COVID-19 diagnosis?  (Choose the highest level of activity you were able to do) | ‌ Vigorous activities, such as running, lifting heavy objects, participating in strenuous sports  ‌ ‌ Moderate activities, such as moving a table, pushing a vacuum cleaner, bowling, or playing golf  ‌ Climbing one flight of stairs  ‌ Walking one block  ‌ Lifting or carrying groceries  ‌ Bathing or dressing yourself |
| **6** | Does your health now limit you in these activities? If so, how much? |  |
|  | Vigorous activities, such as running, lifting heavy objects, participating in strenuous sports | ‌ Yes, limited a lot  ‌ Yes, limited a little  ‌ No, not limited at all |
|  | Moderate activities, such as moving a table, pushing a vacuum cleaner, bowling, or playing golf | ‌ Yes, limited a lot  ‌ Yes, limited a little  ‌ No, not limited at all |
|  | Climbing one flight of stairs | ‌ Yes, limited a lot  ‌ Yes, limited a little  ‌ No, not limited at all |
|  | Walking one block | ‌ Yes, limited a lot  ‌ Yes, limited a little  ‌ No, not limited at all |
|  | Lifting or carrying groceries | ‌ Yes, limited a lot  ‌ ‌Yes, limited a little  ‌ No, not limited at all |
|  | Bathing or dressing yourself | ‌ Yes, limited a lot  ‌ Yes, limited a little  ‌ No, not limited at all |
| **7** | After the COVID-19 diagnosis, how often did you |  |
|  | Have trouble getting things organized during the last 4 weeks? | ‌ Never  ‌ Rarely  ‌ Sometimes  ‌ Often  ‌ Almost always |
|  | Have trouble concentrating on things like watching television program or reading a book during the last 4 weeks? | ‌ Never  ‌ Rarely  ‌ Sometimes  ‌ Often  ‌ Almost always |
|  | Forget what you talked about after a telephone conversation? | ‌ Never  ‌ Rarely  ‌ Sometimes  ‌ Often  ‌ Almost always |
| **8** | Have you had any of the following symptoms over last 4 weeks? Have the symptoms resolved? | ‌ Fever, chills or night sweats – ‌ Resolved  ‌ Loss of smell or taste -- ‌ Resolved  ‌ Fatigue -- ‌ Resolved  ‌ Shortness of breath -- ‌ Resolved  ‌ Chest pain -- ‌ Resolved  ‌ Numbness or tingling -- ‌ Resolved  ‌ Nausea, vomiting, or diarrhea -- ‌ Resolved  ‌ Muscle aches -- ‌ ‌ Resolved  ‌ Rashes -- ‌ Resolved |

**Supplemental Table 1: Relationship of Individual Elixhauser Conditions to PASC**

|  | **Has Condition** | | **No Condition** | |  |
| --- | --- | --- | --- | --- | --- |
| Elixhauser condition | **No PASC** | **PASC** | **No PASC** | **PASC** | **P-Value** |
| AIDS/HIV | 5 | 0 | 588 | 275 | 0.19 |
| Alcohol Abuse | 31 | 8 | 562 | 267 | 0.013 |
| Anemia Deficiency | 103 | 42 | 490 | 233 | 0.44 |
| Rheumatoid Arthritis | 60 | 32 | 533 | 243 | 0.50 |
| Blood Loss Anemia | 22 | 13 | 571 | 262 | 0.48 |
| Cardiac Arrhythmia | 197 | 95 | 396 | 180 | 0.70 |
| Congestive Heart Failure | 99 | 35 | 494 | 240 | 0.13 |
| Chronic Pulmonary Disease | 181 | 95 | 412 | 180 | 0.24 |
| Coagulopathy | 110 | 44 | 483 | 231 | 0.36 |
| Depression | 134 | 70 | 459 | 205 | 0.36 |
| Diabetes with Chronic Complications | 196 | 99 | 397 | 176 | 0.39 |
| Diabetes without Chronic Complications | 189 | 101 | 404 | 174 | 0.16 |
| Drug Abuse | 27 | 26 | 566 | 249 | 0.005 |
| Hypertension, uncomplicated | 384 | 163 | 219 | 112 | 0.28 |
| Hypertension, complicated | 120 | 51 | 473 | 224 | 0.84 |
| Hypothyroidism | 94 | 59 | 499 | 216 | 0.044 |
| Liver Disease | 124 | 62 | 469 | 213 | 0.59 |
| Lymphoma | 23 | 3 | 570 | 272 | 0.025 |
| Fluid and Electrolyte Disorders | 286 | 121 | 307 | 154 | 0.25 |
| Metastatic Cancer | 18 | 13 | 575 | 262 | 0.21 |
| Other Neurologic Disorders | 84 | 38 | 509 | 237 | 0.89 |
| Obesity | 212 | 121 | 381 | 154 | 0.020 |
| Paralysis | 14 | 5 | 579 | 270 | 0.80 |
| Peripheral Vascular Disease | 110 | 42 | 483 | 233 | 0.24 |
| Psychoses | 17 | 7 | 576 | 268 | 1.0 |
| Pulmonary Circulation Disorder | 38 | 16 | 555 | 259 | 0.74 |
| Renal Failure | 168 | 55 | 425 | 220 | 0.009 |
| Solid Tumor without Metastasis | 79 | 38 | 514 | 237 | 0.84 |
| Peptic Ulcer Disease | 23 | 11 | 570 | 264 | 0.93 |
| Valvular disease | 92 | 41 | 501 | 234 | 0.81 |
| Weight Loss | 86 | 33 | 507 | 242 | 0.32 |

N=868 patients with Elixhauser index available. Chi square or Fisher’s exact test, as appropriate.

**Supplemental Table 2A: Multivariable Logistic Regression for Assessing Factors Associated with PASC in Hospitalized Patients**

Covariate OR (95% CI)

Sex

Female 1.29 (0.92-1.80)

Male 1 [Reference]

Age (10 years) 0.98 (0.86-1.11)

Race

White 1 [Reference]

Black 0.80 (0.41-1.56)

Hispanic or Latino 1.004 (0.67-1.52)

Asian 0.78 (0.41-1.48)

Other 0.87 (0.46-1.65)

Unknown 0.59 (0.22-1.64)

BMI 1.024 (1.001-1.049)

Diabetes 1.48 (1.05-2.08)

History of Organ Transplant 0.47 (0.27-0.84)

Payer type

Commercial insurance 1 [Reference]

Medicare 0.95 (0.63-1.45)

Medicaid 0.49 (0.31-0.79)

Other/None 0.87 (0.39-1.91)

Social Vulnerability Index

0-25% 1 [Reference]

25-50% 1.09 (0.69-1.74)

50-75% 0.89 (0.56-1.44)

75-100% 1.04 (0.65-1.66)

Unknown 0.87 (0.38-1.99)

Maximal Exertion before COVID-19

Vigorous 1 [Reference]

Moderate 1.06 (0.69-1.63)

Walking 1 Block 0.86 (0.51-1.45)

Carrying Groceries or Bathing 1.03 (0.44-2.42)

Unknown 0.89 (0.32-2.46)

**Supplemental Table 2B: Multivariable Logistic Regression for Assessing Factors Associated with PASC in Non-Hospitalized Patients**

Covariate OR (95% CI)

Sex

Female 1.29 (0.66-2.52)

Male 1 [Reference]

Age (10 years) 0.85 (0.67-1.08)

Race

White 1 [Reference]

Black 0.70 (0.18-2.79)

Hispanic or Latino 0.60 (0.27-1.32)

Asian 1.11 (0.28-4.28)

Other 0.83 (0.23-3.08)

Unknown 0.45 (0.14-1.40)

BMI 1.02 (0.97-1.07)

Diabetes 0.85 (0.39-1.85)

History of Organ Transplant 0.41 (0.07-2.46)

Payer type

Commercial insurance 1 [Reference]

Medicare 0.93 (0.42-2.05)

Medicaid 0.41 (0.02-11.29)

Other/None 2.15 (0.29-16.06)

Social Vulnerability Index

0-25% 1 [Reference]

25-50% 2.10 (0.88-5.03)

50-75% 1.58 (0.62-4.05)

75-100% 1.43 (0.53-3.86)

Unknown 2.59 (0.56-11.91)

Maximal Exertion before COVID-19

Vigorous 1 [Reference]

Moderate 0.69 (0.32-1.52)

Walking 1 Block 1.01 (0.28-3.64)

Carrying Groceries or Bathing 1.68 (0.29-9.69)

Unknown 0.28 (0.009-8.42)

**Supplemental Figure 1. Survey attrition flow diagram**

**Patients with COVID-19 enrolled in program**

**N=1,296**

**Did not complete surveys N=258**

**Patient completed survey at 30, 60, or 90 days**

**N=1,038 (Hospitalized N=800, Non-Hospitalized N=238)**

**Participants that completed 30 day survey**

**N=849**

**N=849**

**Participants that completed 60 day survey**

**N=763**

**PASC based on 60-day survey, no 90-day survey**

**N=145**

**Participants that completed 60 or 90 day survey**

**N=879**

**Participants that completed 90 day survey**

**N=504**

**PASC**

**N=164**

**Total PASC from 60 or 90 day survey**

**N=309**
